# Association of juvenile idiopathic arthritis with maternal infection: a case control study

**DOI:** 10.1186/s12969-022-00703-9

**Published:** 2022-06-23

**Authors:** Anna Sutton, Sabah M. Quraishi, Susan Shenoi

**Affiliations:** 1 Department of Epidemiology, University of Washington School of Public Health, UW Box # 351619, 3980 15th Ave NE, Seattle, WA 98195 USA; 2 Department of Environmental and Occupational Health Sciences, University of Washington School of Public Health, UW Box # 354695, 4225 Roosevelt Way NE, Seattle, WA 98105 USA; 3 Division of Rheumatology, Department of Pediatrics, Seattle Children’s Hospital and Research Center, University of Washington M/S MA.7.110, 4800 Sand Point Way NE, Seattle, WA 98105 USA

**Keywords:** Juvenile arthritis, Pregnancy, Risk factors

## Abstract

**Objective:**

Maternal infection has been posited as a risk factor for childhood autoimmune disease such as type I diabetes. Given that similar studies in JIA are scant, our objective was to evaluate the association between Juvenile Idiopathic Arthritis (JIA) and maternal infection.

**Methods:**

This case–control study used an existing database that included 1290 JIA cases and 6072 controls matched on birth year. Maternal infection information was obtained from Washington State birth records. JIA diagnosis and categories were confirmed through chart review. Logistic regression was used to calculate adjusted odds ratios (ORs) and 95% confidence intervals (95% CIs).

**Results:**

JIA was not associated with maternal infection (OR = 1.02, 95%CI: 0.8–1.3). There was no association between JIA and maternal infection for persistent oligoarticular, RF negative polyarticular, or enthesitis-related JIA. There was suggestive evidence of an increased association of maternal infection with JIA in females in sex-stratified analysis.

**Conclusions:**

We did not observe an increased risk of JIA in children exposed to maternal infection. Suggestive evidence of differential sex-specific results warrants further study.

## Introduction

Juvenile Idiopathic Arthritis (JIA) is a heterogeneous group of chronic inflammatory arthritides in children ≤ 16 years of age [1]. The etiology is likely multifactorial and complex, involving both genetic and environmental factors.

The developmental origins of disease hypothesis established a potential link between fetal environment and adult onset disease [2]. Prior studies have found maternal prenatal infection is a risk factor for immune function-related health outcomes including asthma, celiac disease, and type 1 diabetes [3–7]. Research on risk of JIA with maternal prenatal infection is scant. A Swedish study reported no association between JIA and prenatal maternal infection, however it was limited to hospitalized children with JIA [8]. Another study linked maternal antibiotic use but not infection itself with systemic JIA diagnosis [9]. Prospectively studying associations of JIA and prenatal maternal infection is difficult due to rarity of JIA diagnosis and recall bias as JIA is often diagnosed several years later in early or late childhood. Hence, we used existing linked medical records and Washington (WA) State birth records to evaluate the association between JIA and maternal infection.

## Methods

### Study design

This case control study used an existing database linking pediatric rheumatology clinic records with WA State birth records [10, 11]. Potential cases were children < 20 years old with JIA, identified by ICD-9 codes, between 1997–2010 at a tertiary care hospital in Washington state (*N* = 1518). Controls were randomly selected in a ratio of 4:1 from remaining birth records for the same years, matched on birth year (*N*= 6072). Further details for the dataset are provided in previous publications [10, 11]. This study was reviewed and approved by WA State and Seattle Children’s Hospital Institutional Review Boards (IRB).

### Juvenile Idiopathic Arthritis

Medical records review of 1518 potential JIA cases eliminated 228 misclassified cases based on ICD-9 codes. The remaining 1290 cases were categorized by International League of Associations for Rheumatology (ILAR) JIA classification as persistent oligoarticular (*n* = 402), extended oligoarticular (*n* = 74), rheumatoid factor (RF) negative polyarticular (*n* = 275), RF positive polyarticular (*n* = 63), systemic (*n* = 75), psoriatic (*n* = 71), enthesitis-related (*n* = 263), and undifferentiated arthritis (*n* = 39). Twenty-eight cases of polyarticular arthritis were categorized as RF unknown because RF titer information was not available.

### Maternal infection

Maternal infection exposure was determined using WA State birth records from 1980–2006 and is considered any infection, including viral or bacterial, indicated on the birth record as occurring during pregnancy or labor and delivery of the index pregnancy.

All infection data on the birth certificate form were indicated as infection present or absent and subsequently combined into a composite variable indicating maternal infection as yes/no for each included case or control. Differences in data availability for specific infections is related to changing requirements for what information was included in various years. The matched case–control by birth year design mitigates any potential confounding by type of data availability. Information for hepatitis B, genital herpes, and syphilis was available for the entire study period (1980–2006). Just over 10% of included cases and controls were born after 2002, so there is limited data for questions asked beginning in 2003 (hepatitis C, group B streptococcus, gonorrhea, chlamydia, other unspecified maternal infection, antibiotics during labor and delivery, and chorioamnionitis during labor). Maternal rubella data was collected from 1980–1991 and thus unavailable for approximately 64% of subjects. Maternal fever during labor and delivery (> 38 degrees Celsius) was collected from 1980–2002 and unavailable for < 28% of cases and controls. Given cases and controls were matched by year, potential bias due to data availability is minimal.

### Statistical analysis and confounders

Odds ratios (OR) and 95% confidence intervals (CIs) for the associations of interest were estimated using logistic regression. Our primary analysis was association of maternal infection exposure and JIA.

Confounders were chosen a priori and identified based on literature review of past JIA studies [8, 10–12]. Our primary analysis adjusted for: infant sex, gestational age (< 37, 37—42, ≥ 42 weeks), maternal race/ethnicity (White, African American, Native American, Asian/Pacific Islander, Hispanic), health insurance billed at delivery as determined from payor status for mother’s hospital delivery (private or Medicaid, used as a proxy for socioeconomic status), and number of older siblings (0, 1, 2 +). Since associations between maternal factors and JIA diagnosis are not well-established, we did a secondary analysis including additional hypothesized confounders, including: maternal age (< 20, 20–34, 35 + years), maternal education (≤ 12, 13—15, 16 + years), c-section delivery (yes, no), and trimester when prenatal care began (1^st^, 2^nd^, 3^rd^, none). As the prevalence of JIA differs between boys and girls for some categories of JIA, we assessed the role of infant sex as an effect modifier using stratified analysis.

We performed exploratory analysis of maternal infection exposure and individual JIA categories when numbers permitted. Maternal infection may impact age at diagnosis of JIA, therefore within cases we also evaluated if there was an association between younger age at diagnosis (< 5 years) compared to older age at diagnosis (≥ 5 years). All analyses were adjusted for the matching variable, birth year, and were conducted using STATA15 software [13].

## Results

As expected, JIA cases were more likely to be female (68%). Mothers of cases were more likely to be white, privately insured, receive prenatal care earlier, and be more educated (Table 1). The distribution of cases and controls for other characteristics was similar (Table 1).

**Table 1:** Infant and maternal characteristics of JIA cases and non-JIA controls

Characteristics	Cases (*N* = 1290)	Controls (*N* = 6072)
	n(%)	n(%)
**Infant**		
Female	871 (68)	2903 (48)
Birthweight (grams)		
< 2500	73 (6)	339 (6)
2500–3999	1067 (83)	4912 (81)
4000 +	148 (11)	803 (13)
Missing	2 (0.2)	18 (0.3)
Gestational age (weeks)		
< 37	82(7)	389 (7)
37–42	1071 (85)	4943 (83)
> 42	104 (8)	588 (10)
Missing	33 (3)	152 (3)
Number of older siblings		
None	541 (43)	2517 (42)
1	442 (35)	1942 (33)
2 +	276 (22)	1474 (25)
Missing	31 (2)	139 (2)
**Mother**		
Age (years)		
< 20	108 (8)	694 (11)
20–34	991 (77)	4636 (76)
≥35	191 (15)	739 (12)
Missing	0	3 (0.05)
Race/Ethnicity		
White	1039 (84)	4638 (78)
Black	24 (2)	233 (4)
Native American	37 (3)	123 (2)
Asian/Pacific Islander	59 (5)	402 (7)
Hispanic	79 (6)	526 (9)
Missing	52 (4)	150 (2)
Education (years)		
< 13	329 (42)	1731 (49)
13 to 15	189 (24)	927 (26)
≥16	265 (34)	888 (25)
Missing	507 (39)	2526 (42)
Married	1032 (80)	4469 (74)
Missing	2 (0.2)	26 (0.4)
Trimester prenatal care began		
1^st^	1017 (86)	4591 (81)
2^nd^	138 (12)	897 (16)
3^rd^	31 (3)	175 (3)
Missing	104 (8)	409 (7)
Cesarean section	277 (22)	1151 (19)
Missing	1 (0.1)	5 (0.1)
**Other**		
Medicaid or Medicare billed for labor and delivery	284 (26)	1789 (37)
Missing	195 (15)	1240 (20)
Residence		
Urban	820 (76)	3787 (77)
Rural	252 (24)	1127 (23)
Missing	218 (17)	1158 (19)

In our primary analysis (Table 2), there was no significant difference in the odds of being diagnosed with JIA based on maternal infection exposure (OR = 1.02, 95%CI: 0.78 to 1.34). When stratified by infant sex, the odds of being diagnosed with JIA were higher for females (OR = 1.12, 95%CI: 0.79 to 1.57) than males (OR = 0.85, 95%CI: 0.53 to 1.37), but this result was not statistically significant (interaction *p* = 0.39). Secondary analysis including additional adjustment variables showed no evidence of a significant difference in odds after correcting for the additional factors (OR = 0.95, 95%CI: 0.68 to 1.33) (Table 2).

**Table 2:** Associations of maternal infection with JIA

	**N**	**OR**	**95%CI lower**	**95%CI upper**	***P*-value**
**Primary adjustment model**^**a**^	5269	1.02	0.78	1.34	0.89
**Secondary adjustment model**^**b**^	3438	0.95	0.68	1.33	0.68
**Stratified by infant sex**^**c**^					
Female	2726	1.12	0.79	1.57	0.39
Male	2543	0.85	0.53	1.37	
**Stratified by JIA category**					
Polyarticular RF-	275	1.17	0.71	1.92	0.54
Persistent oligoarticular	402	1.05	0.70	1.59	0.81
Enthesitis-related	263	0.79	0.40	1.56	0.50
**Age at diagnosis**					
JIA diagnosis < 5 years of age compared to diagnosis ≥ 5 years of age		1.27	0.69	2.34	0.45

^a^ Adjusted for infant sex, gestational age, number of older siblings, Medicaid coverage for labor and delivery, maternal race, and birth year

^b^Adjusted for primary adjusters and maternal education, trimester of first prenatal care, and cesarean section delivery

^c^Stratified by infant sex and adjusted for primary adjustors from above

For exploratory analysis of maternal infection among specific categories of JIA, only RF-negative polyarticular JIA, persistent oligoarticular JIA, and enthesitis-related arthritis had sufficient data for analysis. Due to concerns about the stability of estimates, we did not report results from analyses in which cell count was less than 10. No statistically significant increase or decrease in ORs were found among JIA category-specific ORs with maternal infection (RF-negative polyarticular OR = 1.17, 95%CI: 0.71 to 1.92; persistent oligoarticular OR = 1.05, 95%CI: 0.70 to 1.59; enthesitis-related OR = 0.79, 95%CI: 0.40 to 1.56). We created a larger “polygo” category, combining persistent oligoarticular, extended oligoarticular, and RF-negative polyarticular arthritis and amongst this “polygo” category there was also no significant association (OR = 1.09, 95%CI: 0.80 to 1.51) [14].

When compared with children who were diagnosed with JIA at ≥ 5 years, those diagnosed at < 5 years were 1.27 times as likely to have been exposed to maternal infection (OR = 1.27, 95%CI: 0.69 to 2.34)(Table 2).

## Discussion

Our study using an existing cohort from North America found no association between exposure to prenatal maternal infection and subsequent JIA diagnosis. This is consistent with the findings by Carlens, et al. that found no association between maternal infection and JIA diagnosis among hospitalized children in Sweden [8]. Carlens, et al. used a convenience sample of hospitalized JIA cases and could potentially have underestimated risks as JIA is primarily managed in the ambulatory setting. Kindgren, et al., reported that antibiotic use during pregnancy (a potential surrogate marker of infection) was associated with diagnosis of systemic JIA [9]. In our study, we were not able to evaluate antibiotic use or other treatment to determine how results compare with results for infection.

While our analysis of exposure to maternal infection during pregnancy did not find a statistically significant association between infant sex and JIA, in females there is suggestion of an increased odds and in males there is suggestion of a decreased odds of JIA with maternal infection. This differential direction warrants further study. There was some suggestive evidence in our data that the RF-negative polyarticular category of JIA may be related to maternal infection (Table 2). However, our study may have been underpowered to detect an association. Future studies focused on this category or specific markers of this category may be warranted.

The classification of JIA is continually evolving and ILAR classification for JIA include 7 unique categories, each with potentially different etiologies [15]. We had limited ability to conduct category-specific analysis due to small numbers. Evaluation of risk in overall JIA as a single entity given the heterogeneity within this umbrella diagnosis may have potentially attenuated our observed results. Our infection variable was a composite of multiple infection exposures. If a specific type of maternal infection increases the risk of JIA, our study would not have found that link. We were limited given the retrospective nature of the data in the level of detail collected regarding maternal infection by WA State birth records.

While exposure information was recorded prior to JIA onset, it was recorded at the time of labor and delivery which makes it possible that infection earlier in the pregnancy was underreported. Given the design of the study, there is no reason to suspect this potential misclassification of exposure or missing data by year would have differed between cases and controls, but this may have biased the observed odds ratios toward the null. We may have missed some cases of JIA among those who have limited access to care. While care is available for families without resources at the children’s hospital, children with limited access to care may not have had access to regular pediatric care and thus may not have been referred for diagnosis or have been seen by other centers. Mothers of these children may also have lacked access to care during pregnancy and may have been more likely to experience infection during pregnancy. Inclusion of undiagnosed cases in our control group would likely have diluted any observed association between JIA and maternal infection.

A major strength of our study, particularly since JIA is a rare outcome, is that it utilizes a large dataset of JIA cases linked to state birth records. Our exposures were extracted from birth records and not subject to recall bias. Multiple infection variables are included on the birth certificate records, so we were able to broadly capture maternal infections to include in our composite exposure variable. Medical chart review reduced misclassification of JIA cases by ICD-9 codes. Although it is possible that some children with mild JIA may have been treated by their primary care physician or an adult rheumatologist, Seattle Children’s Hospital is the primary location for treatment of JIA in Washington State, so few cases of JIA diagnosed in the state during the timeframe of our study would have been missed.

While childhood infection has been closely examined as a risk factor for JIA, maternal infection has not. This study is an initial exploration of fetal response to infection as a mechanism for JIA. While we did not find an overall association between JIA and maternal infection, our results suggest that future studies focused on the individual categories of JIA and gender related differences should be considered.

## Data Availability

Restrictions apply to the availability of the data provided by the Washington State Department of Health. Data are available from the authors upon reasonable request and with permission of Washington State Department of Health.

## Abbreviations

JIA: Juvenile idiopathic arthritis
OR: Odds ratio
CI: Confidence interval
ILAR: International League of Associations for Rheumatology
ICD-9: International Classification of Diseases, 9^th^ edition
